# The Effect of Alcohol Compound on the Solidification of Magnesium Oxysulfate Cement-Boron Mud Blends

**DOI:** 10.3390/ma15041446

**Published:** 2022-02-15

**Authors:** Yuanyuan Liang, Yan Guan, Wanli Bi

**Affiliations:** 1 Institute of Materials and Metallurgy, University of Science and Technology Liaoning, Anshan 114031, China; asbwl@126.com; 2 Research Institute of Keda Fengchi Magnesium Building Materials, Anshan 114031, China

**Keywords:** magnesium oxysulfate cement-boron mud blends, KH550, mechanical strength, microstructure, solidification

## Abstract

At present, the utilization of boron resources in China is increasing, and the problem of boron tailing pollution is becoming increasingly serious. To fundamentally solve the problem of boron tailing, many scholars at home and abroad have mainly studied the curing effect in terms of compressive strength, and little research has been carried out into the solidification effect and hydration products. This study explored the effects of adding different alcohol-based modifiers on the hydration products of magnesium oxysulfate cement-boron mud mixture, the microstructure, physical properties and curing effects of the samples. The results show that magnesium oxysulfate cement is beneficial to the solidification of boron in boron mud due to its low-alkali. Adding an alcohol-based modifier can increase the compressive strength of magnesium oxysulfate cement-boron mud blends. After adding acrylic acid and D-Mannitol, the 28-day compressive strength of the sample increased by 44.7 MPa. The blending of alcohol-based modifiers has a very good effect on the curing of boron in the whole system.

## 1. Introduction

According to statistics, the world’s boron resource reserves in 2015 were approximately 380 million tons [1]. The main producers of boric acid and borax in the world are Turkey, the United States, China, Chile and Russia [2]. Boric acid and borax are important primary products with high output values and large outputs. Their production plays a key role in the boron production industry in China [3]. Under low-concentration conditions, boron is also an essential trace element for plant growth [4]. Boron compounds can be used to make adhesives, detergents, flame retardants and other products and can be used as pesticides and fertilizers in agriculture [5,6]. However, in the process of producing boric acid from boron ore, boron mud, a moist waste, is produced. The production of boron mud increases the accumulation of boron content in the soil, and the physical, chemical and biological properties of the soil also change accordingly [7]. In addition, studies have shown that surface water can mix with soil through groundwater and form complexes with copper, plumbum, Ni and Cd ions. These complexes are more toxic than heavy metals [8]. On the other hand, the high concentration of boron in the ground damages the plants in the ground and causes groundwater pollution [9]. Therefore, the production of boron mud has many negative effects on the environment. It is necessary to find a harmless method to dispose of boron mud.

Solidification/stabilization technology has been widely used to improve the mechanical properties of soil and reduce the mobility of pollutants [10]. The process of solidification and stabilization includes chemical stabilization and physical fixation, where physical fixation refers to the fixation of contaminants in the entire matrix through an interaction between waste and adhesive [11]. Studies have shown that the solidification peak temperature of phenolic resin shifts to a low temperature due to the addition of boron, and the activation energy of the solidification reaction of the phenolic resin is also reduced [12]. The addition of boron makes the solidification reaction easier, so boron can accelerate the solidification of phenolic resin [12]. In addition, ordinary Portland cement is a kind of binder for solidifying and stabilizing hazardous waste and is widely used because of its low cost and high efficiency [13]. The use of cementitious materials to transform hazardous wastes into a solidified body with a better structure can reduce the risk of redissolution of harmful substances in hazardous wastes.

Ordinary Portland cement-based binders can be precipitated by metal hydroxides, physically/chemically fixed in calcium silicate hydrate gels, and can also be used to treat pollutants by the incorporation of secondary formed minerals [14]. However, the production process for ordinary Portland cement is related to a high carbon footprint (660–820 kg of carbon dioxide per ton). The production of ordinary Portland cement emits a large amount of carbon dioxide and can aggravate global climate change [15].

Magnesium oxysulfate cement is a new type of magnesium-based cement produced by the reaction of light-burned magnesia and magnesium sulphate [16]. Unlike ordinary Portland cement production (sintering at 1450 °C), the magnesium-rich minerals of the ocean or salt lake can be wet processed to produce magnesium sulphate and magnesium oxide, and light-burned magnesium is produced by calcination of magnesia ore at 650–800 °C [17]. Magnesium sulphate cement is considered to be a low-carbon consolidation material. The reason for this is that compared with ordinary Portland cement production at 1400 °C, this process reduces carbon dioxide emissions during the production process [18]. It is worth noting that the addition of organic citric acid can promote the formation of needle-shaped crystals composed of 5Mg(OH)_2_·1MgSO_4_·7H_2_O (517 phase) in magnesium oxysulfate cement, which changes the original void structure and reduces oxidation [19]. In addition, MgO-based conjugates have advantages over ordinary Portland cement-based conjugates. Its advantage lies in its moderate pH range (approximately 9–10) and good compatibility with pollutants [20]. However, the pH of brucite is approximately 10.5 [21], and most toxic ions have a low solubility [22]. Therefore, magnesium oxysulfate cement can be regarded as a good solidification material. However, there are few studies evaluating boron solidification dissolution in boron mud. Therefore, this paper studies magnesium oxysulfate cement as a solidification material. Fortunately, previous studies have shown that boron exits in the form of a Lewis acid, mainly in the form of unseparated boric acid at low pH or as borate ions at high pH [23]. Chelating resin has a strong attraction to boron due to its hydroxyl group and does not interact with other inorganic parts. At the same time, hydroxyl groups can form different esters with boric acid and rapidly separate by releasing protons to form borate complexes [24].

Therefore, boron is usually removed by the anion exchange resin method. Studies have shown that there are lipids in the sap of plants, and the solidification performance of magnesium oxysulfate cement is improved due to the presence of these lipids [25].

There are few studies on the solidification of boron by magnesium oxysulfate cement and organic modifiers and the evaluation of boron leaching after solidification. In this study, the phase composition, micromorphology and leaching characteristics of boron by magnesium oxysulfate cement with organic modifiers are discussed.

## 2. Experimental

### 2.1. Materials

**Figure 1:**
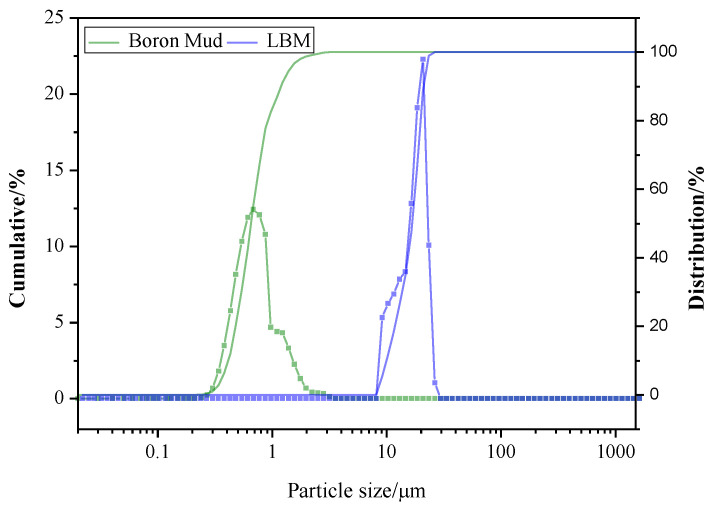
The particle size distribution obtained by a laser detector.

The light-burned magnesia used in this experiment was produced by the China Haicheng Huafeng Group (Liaoyang, China) by calcining magnesite at 800 °C for 1 to 2 h. The content of active MgO (a-MgO) was determined by the hydration method to be approximately 62.10%. Boron mud was obtained from the Dalian Jinma Group (Dalian, China) as a byproduct of boric acid and borax. The chemical compositions of both are listed in Table 1. BT-9300s BETTER laser detector was used to determine the particle size distribution, as shown in Figure 1. Ninety-nine percent pure magnesium sulphate heptahydrate crystals were obtained for experiments. Analytical acrylic acid (C_3_H_4_O_2_) and silane coupling agent—KH550 (analytical pure, Jiangsu Chenguang Group (Jiangning, China)) were used; Glycerol (C_3_H_8_O_3_, Analytical Pure, Damao Chemical Reagent Factory, Tianjin, China), D-Mannitol (C_6_H_14_O_6_, analytical pure, Sinopharm Chemical Reagent Co., Ltd., Shanghai, China) were used as additives for magnesium oxysulfate cement; these modifiers are used to modify magnesium oxysulfate cement and solidify boron ions. Citric acid (CA analytical pure, Ruijinte Chemical Co., Ltd., Tianjin, China) was added to each group of samples to make the 517 phases in the system grow better.

### 2.2. Specimen Preparation

Epsom salt has low molecular solubility, so one should first prepare a solution with a water to magnesium sulphate molar ratio of 20, and the ratio of magnesium sulphate to light-burned magnesia is maintained at 8 to prepare magnesium oxysulfate cement. Citric acid with a mass fraction of 0.3% by weight of LBM is added to better form the 517 phase [19]. To study the immobilization mechanism for boron, four analytical reagents, KH550, acrylic acid, glycerol and D-Mannitol, were added to the magnesium oxysulfate cement paste. The specific dosage is shown in Table 2. To prepare the magnesium oxysulfate cement paste, first, a mechanical mixer was used to mix magnesium sulphate and organic citric acid at a low speed of 60 r/min for two minutes. Then, other additives required for the experiment were added to the prepared mixed slurry of magnesium oxysulfate cement and boron mud and stirred at a high speed of 300 r/min for two minutes. Next, the mixed slurry was poured into a mould with a size of 40 mm × 40 mm × 40 mm and demoulded after approximately 24 h of curing. Finally, the removed module was placed into a curing box for curing at a humidity of 60 ± 5% and temperature of 25 ± 2 °C. To explore the solidification effect on boron ions for different modifiers under different pH conditions and the effect of different pH changes on this solidification, 50 mL deionized water, 0.001 mol/L NaOH, and 1 mol/L NaOH were used to simulate an environment with a pH of 7, 11 and 14, respectively. The specific mixing method is shown in Table 3.

### 2.3. Testing Methods

The initial setting and final setting times for the samples were determined according to the GB/T1346-2011 test standard. For the compressive strength test, an electronic servo testing machine (Cangzhou Jingwei 300S, Cangzhou, China) was used to measure 7 samples (40 mm × 40 mm × 40 mm). The maximum load of the machine was 300 kN, and the loading speed was 0.6 kN/s [26].

XRD test (XRD, Malvern Instruments Limited and PANalytical B.V. Malvern, UK) samples were passed through a 74 μm sieve (Pass λCu = 0.15418 nm, tube pressure: 40 kV, tube flow: 40 mA, start angle = 5°, end angle = 70°, step length of 0.026°, 10 s counting time per step) to determine the crystalline composition of the magnesium oxysulfate cement.

A scanning electron microscope (SEM) (ZEISS SIGMA HD, Jena, Germany) was used to observe the microscopic morphology of the hydration products of an Au-coated sample. The sample was cut into a shape with a diameter of approximately 5 mm and a height of approximately 1 mm. An Agilent Technologies Cary 630 FT-IR Fourier infrared spectrometer was used to measure the functional groups present in the magnesium oxysulfate cement sample at wavenumbers ranging from 450 cm^−1^ to 4000 cm^−1^.

Thermal analysis was performed for the hydrated sample (STA449F3, Netzsch, Germany), and the powdery sample heated in the temperature range of 10 °C to 1100 °C in a 50 mL/min nitrogen gas stream was analysed.

Boron leaching in the magnesium oxysulfate cement sample was measured by the ICP-MS method (Agilent 7500c inductively coupled plasma mass spectrometer, Agilent Technologies Co. Ltd., Santa Clara, CA, USA).

X-ray photoelectron spectroscopy (XPS) was used to determine the presence of oxides and organic layers in the first few nanometres of the surface and the elemental composition [27]. XPS analysis was performed using a TFA physical Electronics Inc. Spectrometer equipped with a hemisphere analyser. All spectra were collected using a monochromatic AlKα X-ray source (1486.6 eV), and the diameter of the analysis area was 400 µm.

## 3. Results

### 3.1. Effect of Modifiers on the Setting Time for Magnesium Oxysulfate Cement-Boron Mud Blends

**Figure 2:**
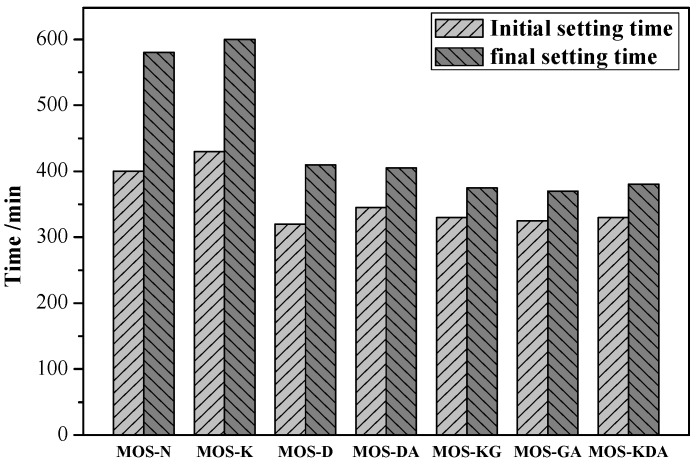
Initial and final setting times for magnesium oxysulfate cement-boron mud blends mixed with different modifiers.

Setting time is one of the most important aspects to describe the applicability of magnesium oxysulfate cement. Figure 2 shows the influence of different modifiers on the setting time of the samples. The addition of boron mud prolongs the initial and final setting times of the samples. Sarkar [28] studied the retardation kinetics for the setting reaction and found that boric acid helps to form a polymer coating on the surface of the MgO particles and hinders the setting of the cement, which can be used as a retarder. Furthermore, Wagh and Jeong [29] found that when boric acid is added to the phosphate solution, magnesite is formed on the MgO grains, gradually covering them and preventing their dissolution. This is consistent with the result that the initial and final setting times for MOS-N are extended to 397 min and 573 min, respectively, after adding boron mud in this experiment. The initial and final setting times for the sample with the modifier are significantly shorter than those for sample MOS-N. Compared with MOS-N, the initial setting time for MOS-D~MOS-KDA is shortened by 82 min, 55 min, 52 min and 67 min, respectively. Compared with MOS-N, the final setting time is shortened by 165 min, 171 min, 185 min and 193 min, respectively. Compared with MOS-N, the initial and final setting times for MOS-K are extended: the initial setting time is extended by 39 min, and the final setting time is extended by 18 min. This is because the addition of polyol leads to bonding of the hydroxyl and magnesium ions to promote the hydration rate.

### 3.2. Influence of Modifier on the Mechanical Properties of Magnesium Oxysulfate Cement-Boron Mud Blends

**Figure 3:**
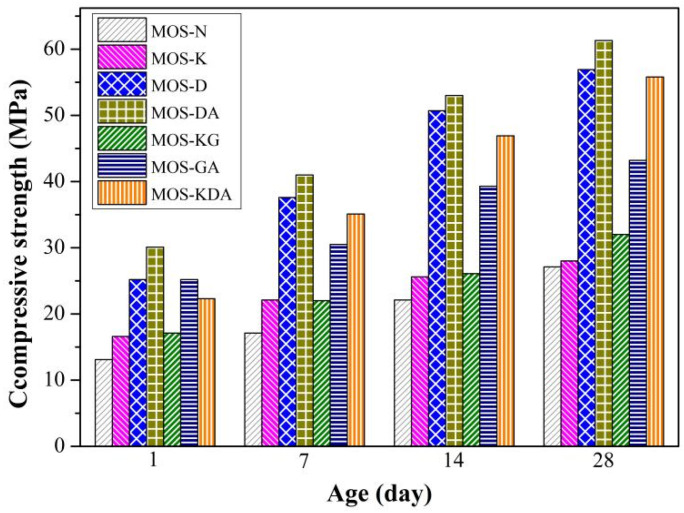
Compressive strength of magnesium oxysulfate cement with different modifiers.

Figure 3 shows the compressive strength for magnesium oxysulfate cement-boron mud blends at 3 days, 7 days, 14 days and 28 days under the control sample and different modifiers. With increasing hydration time, the compressive strength of the specimens shows an increasing trend. Compared with the 1-day compressive strength, the 28-day compressive strength of each group of samples increased by 24.3 MPa, 9.6 MPa, 26.6 MPa, 44.7 MPa, 22.6 MPa, 13.1 MPa and 26.5 MPa, respectively, since the modifier added in MOS-D, MOS-DA and MOS-KDA is an organic compound containing −OH or −COOH. Therefore, the addition of a modifier can improve the mechanical properties of magnesium oxysulfate cement [25]. However, the strength of KH550 is not significantly increased. This is because the molecular formula of the silane coupling agent KH550 is NH_2_(CH_2_)_3_Si(OC_2_H_5_)_3_, which is alkaline after hydrolysis in water [29]. It is a hydrophobic group for magnesium oxysulfate cement surface coupling. This will lead to a weakening of the dispersibility of the entire system of magnesium oxysulfate cement, which easily forms agglomerates, which will negatively affect the mechanical properties of magnesium oxysulfate cement [30].

### 3.3. The Influence of Different Modifiers on the Hydration Products of Magnesium Oxysulfate Cement-Boron Mud Blends

**Figure 4:**
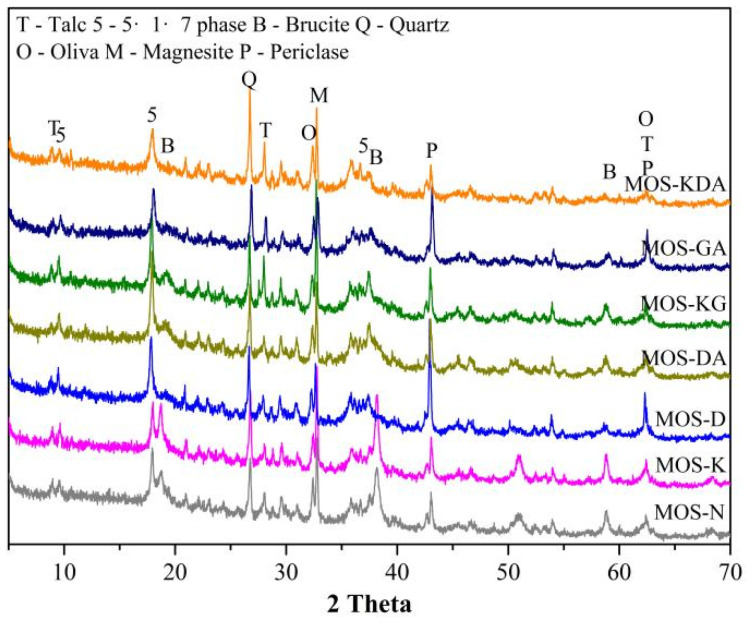
The XRD curves of magnesium oxysulfate cement-boron mud blends after air solidificating for 28 days adding different modifiers.

Figure 4 shows the XRD curves of magnesium oxysulfate cement-boron mud blends after air solidificating for 28 days when adding different modifiers and compares the content of various mineral phases in magnesium oxysulfate cement-boron mud blends. The hydration products for the samples are mainly brucite, quartz, magnesite, 517 phase and olivine. With the addition of D-mannitol, the content of the 517 phase becomes significantly higher than that of phase MOS-N, and the content of brucite is reduced. Accompanied by the simultaneous addition of acrylic acid and D-mannitol, 517 phase content also increases, and the addition of modifiers inhibits the growth of brucite, resulting in a significant decrease in the brucite phase content compared with MOS-N. When different alcohol groups are added to magnesium oxysulfate cement-boron mud blends, more 517 phases are produced [31,32,33], indicating that the addition of modifiers is beneficial to the growth of boron mud in the system, and, at the same time, it also inhibits the formation of brucite. However, when KH550 is added, it is found that the brucite content is higher than that of the control. Also, the sample strength of the added D-mannitol is generally high because the addition of D-mannitol produces more 517 phases.

**Figure 5:**
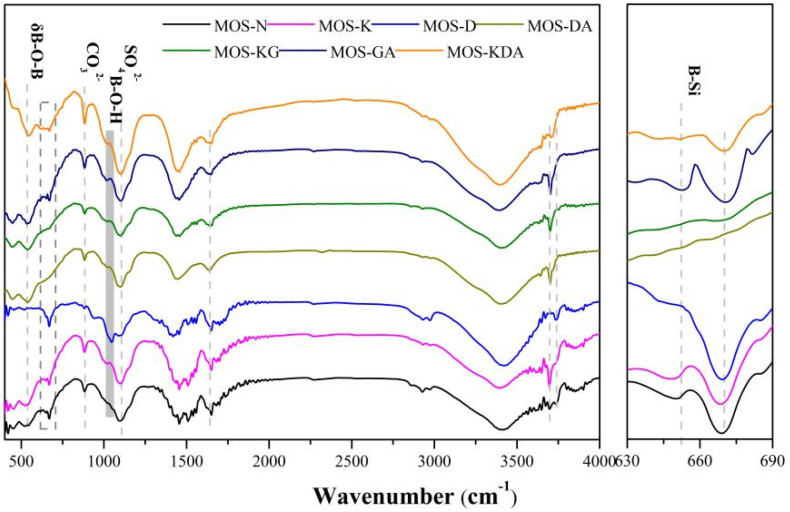
FTIR absorption spectra for control and mixed samples with different modifiers.

Figure 5 shows the FTIR absorption spectra for the control and the samples mixed with different modifiers. Each group of samples of 890 cm^−1^ absorption band is produced by CO_3_^2−^ bending vibration of 1080 cm^−1^ in the absorption band produced by SO_4_^2−^ from the 517 phase stretching vibrationMOS-D~MOS-KDA. The absorption band formed at 1150 cm^−1^ is caused by B-OH bond stretching vibration, and the absorption band MOS-K, MOS-KG and MOS-KDA formed at 650 cm^−1^ is caused by B-O-Si stretching vibration. This shows that the addition of modifiers can effectively make the B ions in the system form relatively stable chemical bonds. Absorption bands are observed at 1150 cm^−1^, which are generated by B-OH bond stretching and vibration [12]. The difference compared to other alcohol bases is that MOS-K shows an absorption band at 650 cm^−1^ due to B-O-Si bond stretching and vibration [34].

**Figure 6:**
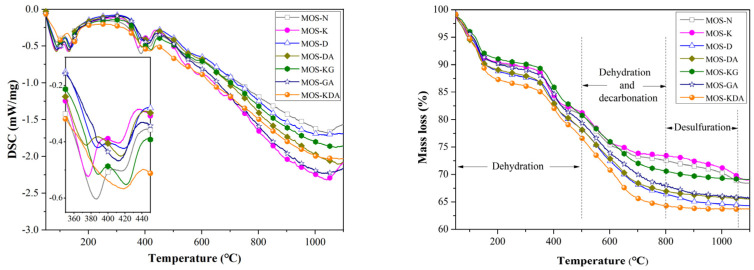
TG-DSC curves for the control and mixed samples with different modifiers.

Figure 6 shows the TG-DSC curves for the control group and the samples mixed with different modifiers. The TG curve for this system mainly shows the decomposition process for the 517 phases of the hydration product and brucite. The TG curve shows that the weight loss from MOS-N to MOS-KDA can be divided into two stages. This first stage of weight loss before ~200 °C corresponds to two absorption peaks in the DSC curve. The main reason for the weight loss in this stage is the dehydration of the 517 phase to produce 5Mg(OH)_2_·MgSO_4_ [35] (the decomposition reaction is shown in Equations (1) and (2)). The second stage of weight loss occurs over the temperature range of 350–650 °C. This stage corresponds to two absorption peaks in the DSC curve. As the temperature continues to rise, 5Mg(OH)_2_·MgSO_4_ continues to decompose to produce MgO, SO_3_ and H_2_O [34] (the specific reaction is shown in Formulas (3) and (4)).

In the TG curve, compared with MOS-K, MOS-N shows more weight loss, indicating that KH550 has an inhibitory effect on the formation of the 517 phase in the system. With the addition of glycerol (MOS-GA), the production of the 517 phase in MOS-K is improved, but there is still a gap compared with the production of the 517 phase in MOS-D, MOS-DA, MOS-GA and MOS-KDA. This also explains why the compressive strength of MOS-GA is only higher than that of MOS-N and MOS-K. The weight loss of MOS-D, MOS-DA, MOS-GA and MOS-KDA in the TG curve is not much different, and the reason for the difference in strength will be explained in the subsequent microstructure.

(1) 5Mg(OH)_2_·MgSO_4_·7H_2_O(s)→5Mg(OH)_2_·MgSO_4_·4H_2_O(s) + 3H_2_O         
81 °C

(2) 5Mg(OH)_2_·MgSO_4_·4H_2_O(s)→5Mg(OH)_2_·MgSO_4_(s)·4H_2_O(g)          
131 °C

(3) 5Mg(OH)_2_·MgSO_4_(s)→5MgO·MgSO_4_(s) + 5H_2_O(g)              
372 °C

(4) 5MgO·MgSO_4_(s)→6MgO(s) + SO_3_(g)                    
955 °C

### 3.4. The Effect of Alcohol Groups on the Microscopic Morphology of Magnesium Oxysulfate Cement-Boron Mud Blends

**Figure 7:**
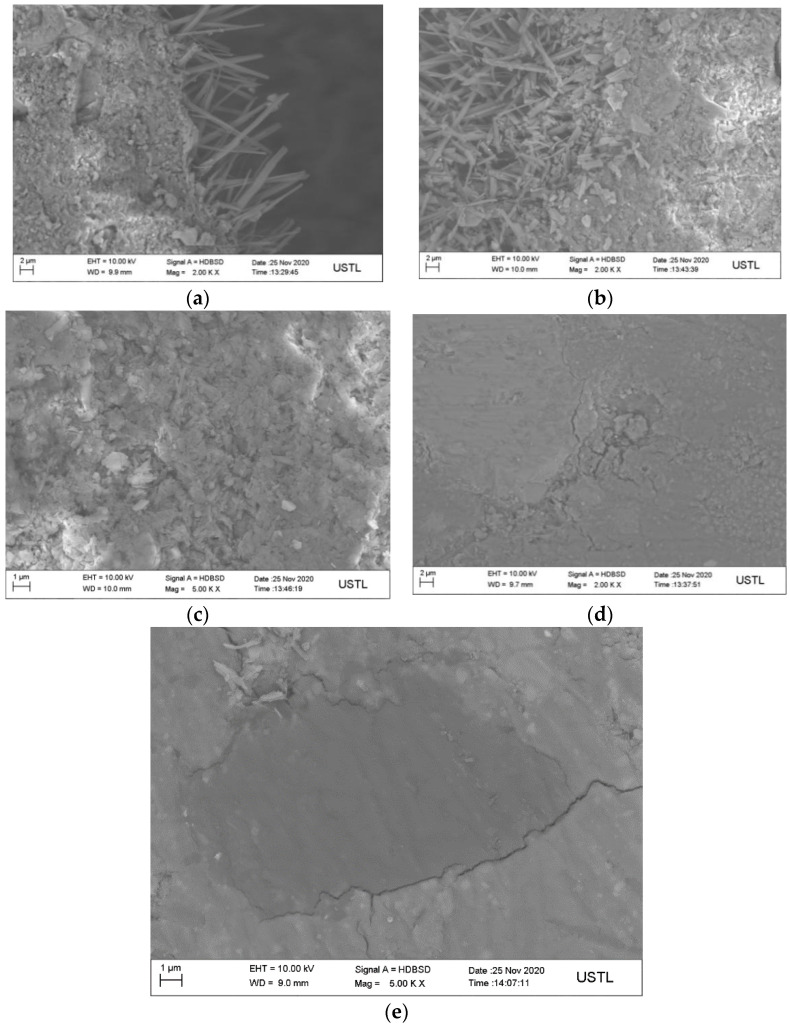

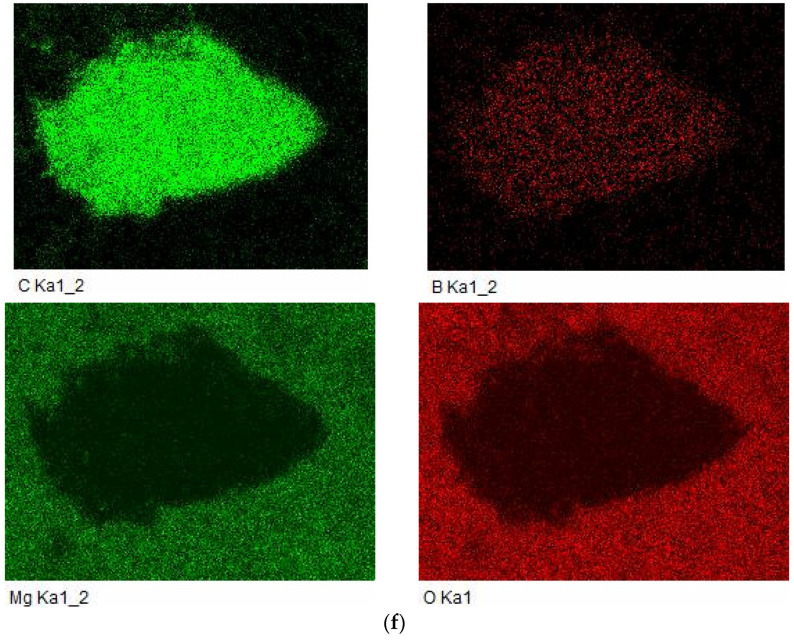
SEM for the control group without modifiers:(**a**) pore of sample MOS-KDA; (**b**) pore of sample MOS-N; (**c)** matrix MOS-KDA; (**d**) matrix MOS-N; (**e**) magnesium oxysulfate matrix; (**f**) EDS spectrum of magnesium oxysulfate matrix.

Figure 7 shows the SEM images of MOS-N mixed with MOS-KDA. As shown in Figure 7a,b, the whiskers in the pores of sample MOS-KDA develop better than those of sample MOS-N. Figure 7c,d show that short columnar crystals appear in matrix MOS-KDA. The ratio of the number of Mg to S atoms detected by EDS is 6:1, which proves that the short columnar 517 phase is formed in matrix MOS-KDA. There are a large number of lamellar crystals in matrix MOS-N, and the Mg to O atomic ratio was determined by EDS to be 1:2, which confirms that a large amount of lamellar magnesium hydroxide is generated in matrix MOS-N. At the same time, as shown in Figure 7e,f, in a magnesium oxysulfate matrix, the presence of boron is identified through the EDS spectrum, and a large amount of carbon is found at the boron position, showing that boron is wrapped in carbon. This proves that carbon can adsorb boron and further proves that the addition of alcohol groups can adsorb boron.

**Figure 8:**
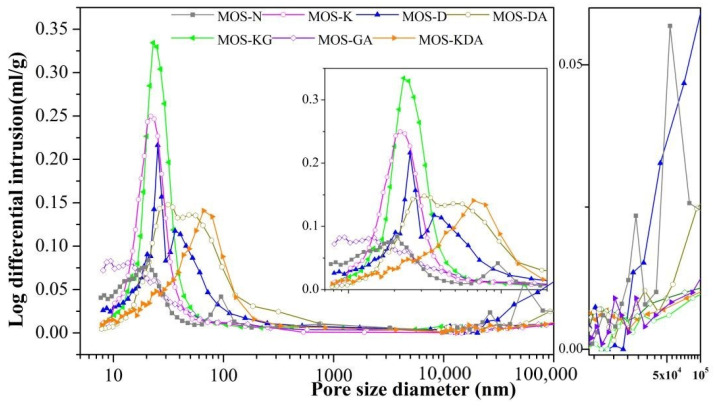
Pore size distribution for samples under different modifiers.

The pore size distribution with different modifiers is shown in Figure 8. Samples MOS-N and MOS-D have many pores above 10,000 nm. The samples for the other groups show little difference, and no large pores are found. However, the compressive strengths of MOS-K, MOS-DA, MOS-KG, MOS-GA and MOS-KDA are quite different. The reason for this may be that with an increasing solidification period, brucite is generated in the original pores, which changes the porosity [36]. The porosities of MOS-D, MOS-DA and MOS-KDA increase. This is due to the larger molecular weight formed by adding D-mannitol, which makes it difficult to dissolve the sample in water, resulting in enrichment. The addition of D-mannitol increases the porosity of magnesium oxysulfate cement-boron mud blends, which can be due to the pores in the system being filled due to enrichment [37]. The structure of the system is affected by the addition of the modifier. Larger pores can result in an increase in the dissolution rate for boron ions, and smaller pores can decrease the leaching rate.

## 4. Study of Different Modifiers on the Solidification Performance of Boron and the Effect of Different pH Values on the Solidification Effect

**Figure 9:**
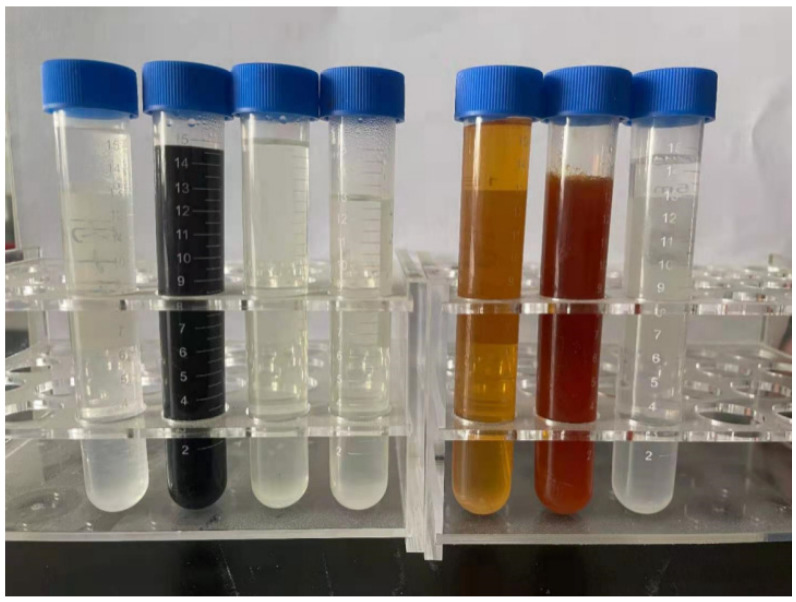
Solidification effect of different modifiers on borax.

Figure 9 shows the experimental results obtained for the solidification effect of different modifiers on borax. The order from left to right is as follows B-GA, B-KDA, B-KG, B-K, B-D, B-DA, B-N. All the samples react violently, and the colour of the samples with D-mannitol added also changes successively. No obvious phenomenon was found to occur in the remaining samples.

**Figure 10:**
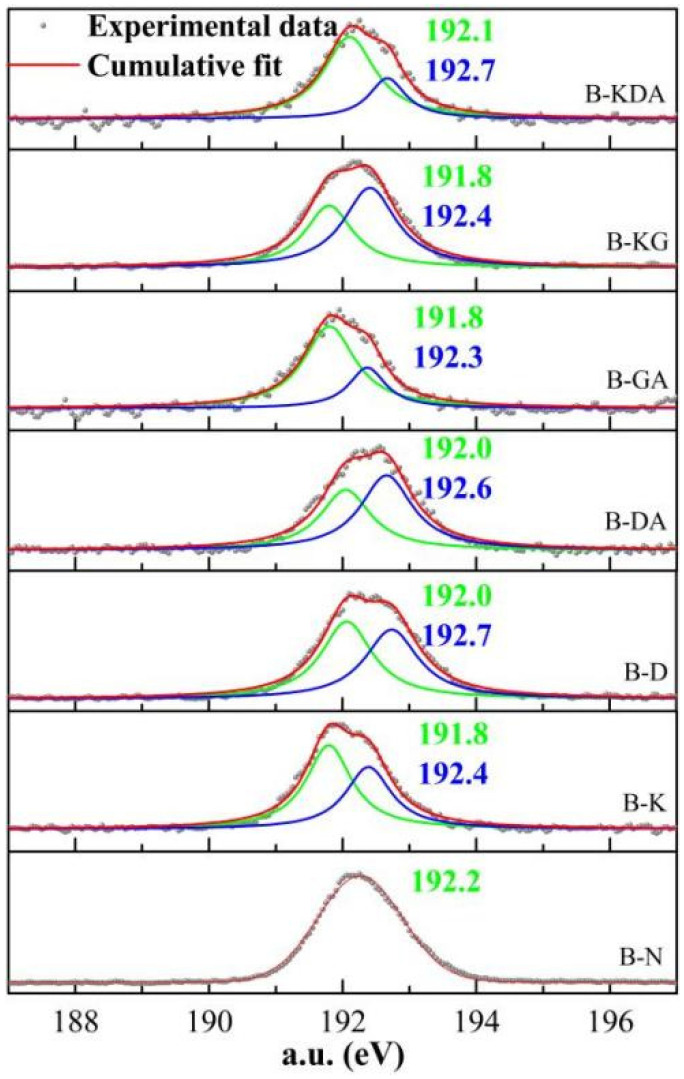
XPS for different samples.

Figure 10 shows the XPS images of different samples. The original bond energy of boron in borax is 191.8 eV [38]. The bond energies of boron in the samples doped with different modifiers are shifted, and the bond energy shows an upward trend. The energy of the sample with KH550 increases to 192.4 eV. The deviation in the composition upon adding D-mannitol is more obvious, and the bond energy of boron is increased. This means that the bond energy of boron in the system is increased after adding D-mannitol, and the bond produced by the combination of boron and D-mannitol is more stable, so that D-mannitol has a stronger adsorption capacity for boron in the system and the adsorption effect is better.

**Figure 11:**
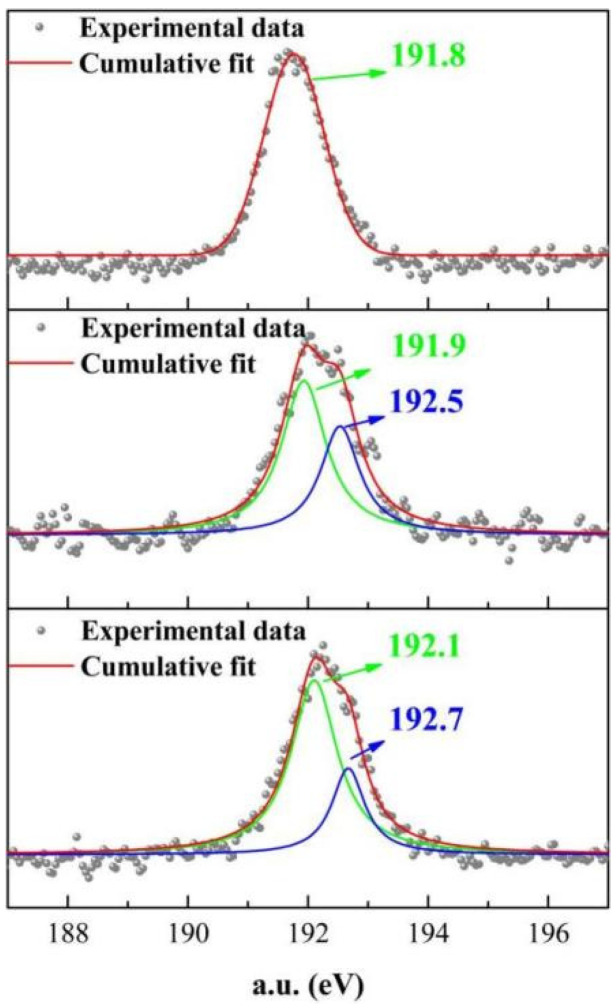
XPS under pH = 7, pH = 11, and pH = 14.

Figure 11 shows the XPS images of MOS-KDA at pH 7, pH 11 and pH 14. The bond energy of boron is different under different pH values. At pH 7, the bond energy of boron ions increases from 191.8 eV to 192.7 eV. For pH 11, the bond energy of boron ions increases from the original value of 191.8 eV to 192.5 eV. At pH 14, the bond energy of boron ions does not change. The bond energy of boron increases and rises at pH 7 and 11. This shows that with increasing pH, the bond energy of boron in the system gradually weakens, a pH of 7 is the most suitable for boron ions to react with other ions in the system, and the solidification effect for boron ions is the best. Since ordinary Portland cement has a higher pH, magnesium oxysulfate cement has a low pH. Therefore, magnesium oxysulfate cement is more suitable for solidifying boron than ordinary Portland cement.

## 5. Effects of Different Modifiers on Boron-Ion Leaching

**Figure 12:**
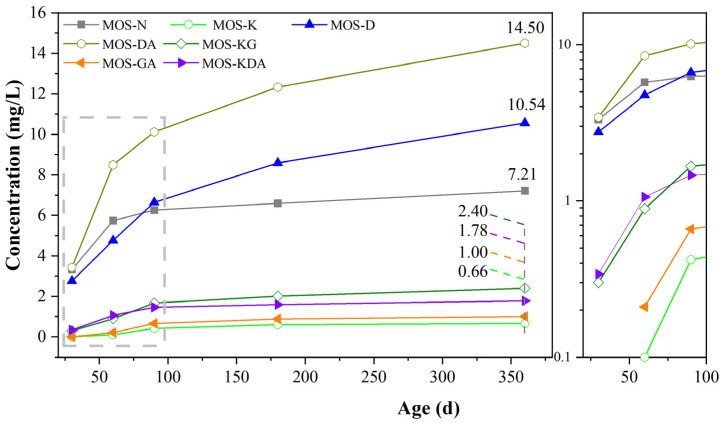
ICP curve for the leaching of boron in magnesium oxysulfate cement-boron mud blends mixed with different modifiers.

Figure 12 shows the ICP curves for boron-ion leaching in magnesium oxysulfate cement-boron mud blends mixed with different modifiers. The curve shows that the leaching rate is increased after the addition of D-mannitol, which is due to the larger porosity, which leads to the infiltration of water. Therefore, the leaching rate for boron increases with the addition of D-mannitol. The leaching rate for the samples containing other modifiers decreases compared with MOS-N. Samples MOS-K, MOS-KG, MOS-GA, MOS-KDA all show a good solidification effect; the leaching rate after immersion for 360 d drops from 7.21 mg/L to 2.4 mg/L, 1.78 mg/L, 1.00 mg/L, 0.66 mg/L, respectively, which conforms to GB5085.3. Therefore, these modifiers can be used for the solidification of boron. The reason for the low leaching rate of MOS-GA is due to the formation of B-OH bond in the magnesium oxysulfate cement-boron mud mixed paste system, while the reason for the lower leaching rate of MOS-K, MOS-KG and MOS-KDA is that the magnesium oxysulfate cement-boron mud B-O-Si bonds are formed in the mixed paste system, which reduces the free boron ion content.

## 6. Conclusions

1. After adding boron mud, the compressive strength of magnesium oxysulfate cement decreases. However, the compressive strength is significantly improved after compounding with D-mannitol, acrylic acid and glycerol. This shows that these modifiers can be used to improve the mechanical properties of magnesium oxysulfate cement-boron mud blends.
2. Adding KH550, acrylic acid, glycerol and D-Mannitol can increase the bond energy of boron and improve the solidification ability towards boron, but after adding D-Mannitol, a large molecular weight structure is formed that increases the porosity; thus, its solidification effect on boron is poor. Although KH550 has the best solidification effect on boron, its strength is low. Consequently, when combining mechanical properties and solidification effects, the use of the compound formula has a better effect.
3. With increasing pH, the solidification effect of magnesium oxysulfate cement on boron is reduced. Therefore, magnesium oxysulfate cement, as a low-alkali cement, is more conducive to the solidification of boron in boron mud than ordinary Portland cement.

## Figures and Tables

**Table 1:** Chemical compositions of light-burned magnesia (LBM) and Boron mud.

Component	Content (wt%)								
	SiO_2_	CaO	MgO	Fe_2_O_3_	Al_2_O_3_	B_2_O_3_	K_2_O	Na_2_O	LOI
Borax Boron Mud	33.79	6.95	23.43	6.00	7.52	2.10	1.40	1.13	17.68
Light-burned Magnesia (LBM)	6.51	1.30	85.08	0.27	0.80				6.04

**Table 2:** Mixing design for magnesium oxysulfate cement paste.

Experiment	Magnesium Oxide	Boron Mud	Citric Acid	KH550	D-Mannitol	Glycerol	Acrylic Acid
MOS-N	1000 g	1000 g	3 g				
MOS-K	1000 g	1000 g	3 g	80 g			
MOS-D	1000 g	1000 g	3 g		80 g		
MOS-DA	1000 g	1000 g	3 g		80 g		40 g
MOS-KG	1000 g	1000 g	3 g	80 g		80 g	
MOS-GA	1000 g	1000 g	3 g			80 g	40 g
MOS-KDA	1000 g	1000 g	3 g	80 g	80 g		40 g

**Table 3:** The ratio of each component under varying pH.

The Serial Number	Borax	Acrylic Acid	D-Mannitol	Glycerol	KH550	Water
B-N	33.9					50
B-K	33.9				80	50
B-D	33.9		80			50
B-DA	33.9	40	80			50
B-GA	33.9	40		80		50
B-KG	33.9			80	80	50
B-KDA	33.9	40	80		80	50

## Data Availability

Data is contained within the article.
